# Microbiological Spectrum and Antibiotics Sensitivity Pattern of Chronic Suppurative Otitis Media (CSOM) Patients: A prospective study from a Tertiary Care Hospital in Peshawar

**DOI:** 10.12669/pjms.41.1.9992

**Published:** 2025-01

**Authors:** Imran Khan, Muhammad Osama, Ihsan Ullah, Inzimam Ul Haq

**Affiliations:** 1 Imran Khan Assistant Professor, Department of ENT, MTI Khyber Teaching Hospital, University Road Peshawar, Pakistan; 2 Muhammad Osama House Officer, MTI Khyber Teaching Hospital, University Road Peshawar, Pakistan; 3 Ihsan Ullah Postgraduate Trainee, MTI Khyber Teaching Hospital, University Road Peshawar, Pakistan; 4 Inzimam Ul Haq Postgraduate trainee, Department of ENT, MTI Khyber Teaching Hospital, University Road Peshawar, Pakistan

**Keywords:** CSOM, Antibiotic resistance, Antibiotic sensitivity, Pseudomonas aeruginosa, Staph aureus

## Abstract

**Background & Objective:**

Chronic suppurative otitis media is a fatal condition owing to its propensity for intracranial extension. The inadvertent use of antibiotics has led to resistance among causative organisms. The objectives of this study were to determine causative bacteria, their antibiotic resistance and susceptibility patterns, and their response to antibiotics after a one-month follow-up.

**Method:**

It was a prospective study conducted in the ENT department of Khyber Teaching Hospital, Peshawar from June 2023 to March 2024. Ear swabs were collected for microscopy and bacterial culture. Statistical analysis was done using IBM-SPSS-20, and GraphPad Prism.

**Results:**

Data was collected from 113 patients. The mean age of the patients was 22 + 2 years. Out of 113 samples, 97(85.5%) showed growth on culture whereas 16(14%) showed no growth, with 95 (98%) unimicrobial, while 2 (2%) showed polymicrobial growth. Pseudomonas aeruginosa was the commonest bacteria isolated 38(38%), followed by *S. aureus*, *Proteus species*, and *S. saprophyticus*. Gram-positive bacteria were susceptible to Linezolid (80%), followed by Vancomycin (76%), with resistance to Ciprofloxacin (75-84%), Ampicillin (70%), and Ceftriaxone (75-80%). Gram-negative bacteria showed susceptibility to carbapenem (50-100%) and Piperacillin/Tazobactam (50% to 93%), with resistance to Ciprofloxacin (50-87%), Ceftriaxone (87%), and Amoxicillin/Clavulanate (72-90%). After one month of follow-up, 82% of patients showed improvements, with noncompliance significantly associated with the persistence of symptoms (p<0.01).

**Conclusion:**

Moderate to high resistance against ciprofloxacin, third-generation cephalosporin, ampicillin, amoxicillin plus clavulanate, and clindamycin is an eye-opener. Our results underscore the critical need for judicious administration of empirical antibiotics.

## INTRODUCTION

Chronic suppurative otitis media (CSOM) is a common infection of the middle ear and can be a fatal condition if left untreated owing to its propensity for intracranial extension. The two most common organisms involved in its causation are Pseudomonas aeruginosa and Staphylococcus Aureus.^1^ The condition is dealt with antibiotics, combined with other modalities of treatment like topical application of antiseptics and aural toilet.^2^ But over the years, the inadvertent administration of antibiotics in Chronic Suppurative Otitis Media (CSOM) has resulted in the development of resistance to the previously effective treatment among the causative organisms.

Studies have isolated strains of pseudomonas aeruginosa resistant to aminoglycosides and penicillin.^3^ Left unaddressed; the issue can have health-related as well as economic consequences. The identification of the right antibiotics with acceptable effectiveness against the causative organism can halt the progression of antibiotic resistance and can prevent the injudicious use of antibiotics. A study in West Bengal found Staphylococcus Aureus sensitive to Linezolid, vancomycin, and ciprofloxacin.^4^ The same study revealed Polymyxin B, meropenem, Cefoperazone plus sulbactam and ciprofloxacin to be highly effective against Pseudomonas Aeruginosa.^4^

The problem is also very common in Pakistan with the initiating event usually an upper respiratory tract infection that irritates followed by inflammation of the middle ear mucosa resulting in CSOM. The disease usually follows a prolonged course because of frequent recurrent episodes of upper respiratory tract infections, inadequate treatment, and medication noncompliance, leading to the emergence of resistance among the pathogens^5^. The same two organisms were found to be the most prevalent ones in Pakistan as well. The purpose of this study is to determine the causative bacteria, their antibiotic resistance and susceptibility patterns, and their response to antibiotics over a two months follow-up.

## METHODS

It was a prospective study conducted in the ENT department of KTH, Peshawar, Pakistan, from June 2023 to March 2024 among one hundred and thirteen (113) patients with CSOM.Patients with persistent or intermittent discharge for over 12 weeks, in patients who have not used antibiotics locally or systemically before collection of the sample were selected, whereas patients with antibiotics use before sample collection, acute otitis media, cholesteatoma, patients with concomitant external ear infection, and those who were not ready for voluntary participation were excluded from the study.

**Fig.1:**
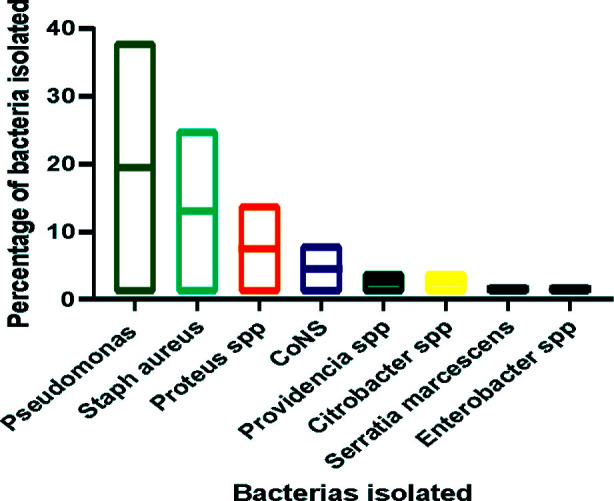
Distribution of bacteria isolated from CSOM patients.

### Ethical Approval:

It was obtained from The Institutional Research and Ethical Review Board (IREB) of Khyber Medical College (KMC), Peshawar (Ref no.105/DME/KMC. Dated June 21, 2023).

To collect samples for microscopic examination and bacterial culture, an aseptic swab was used for the collection of the pus sample from both ears. Under a good light source, first, the external ear canal was cleaned with the alcohol swab, then with the help of a speculum, the cotton tip swab was introduced into the external auditory canal with caution to avoid contact with the surrounding skin, followed by the stick placement in the tube and placement of the cap. The swab was shifted promptly to the Microbiology laboratory. To prepare each sample, a uniform and thin smear was created and left to air-dry. Gram staining was then used to observe the gram reaction, morphology, and arrangement of bacteria under a microscope. All the samples were then introduced into different media including nutrient agar, blood agar, and MacConkey’s agar media (Oxoid Ltd, Hampshire, UK), and kept in an aerobic condition at 37°C for 18-20 hours.

The bacteria were identified based on their microscopic features such as gram reaction, shape, and arrangement, and by taking into account their colony characteristics (including colony morphology, and hemolysis), and biochemical tests (including urease, citrate, and triple sugar iron). To determine the sensitivity pattern of different bacterial pathogens, we used a modified Kirby Bauer disc diffusion method, by following the guidelines recommended by the Clinical and Laboratory Standard Institute (CLSI 2024). For each isolate, we prepared a colony suspension (by using distilled water or even directly by using swab stick) and inoculated it into Mueller-Hinton agar (Oxoid Ltd, Hampshire, UK). We selected commercially available antibiotic discs (Oxoid Ltd, Hampshire, UK) and placed them onto the media, which we then incubated at 37°C for 24 hours. The quality of the media was checked by checking its pH, and expiry date, and checking for any growth in the media without any inoculation over 24 hours.

**Fig.2:**
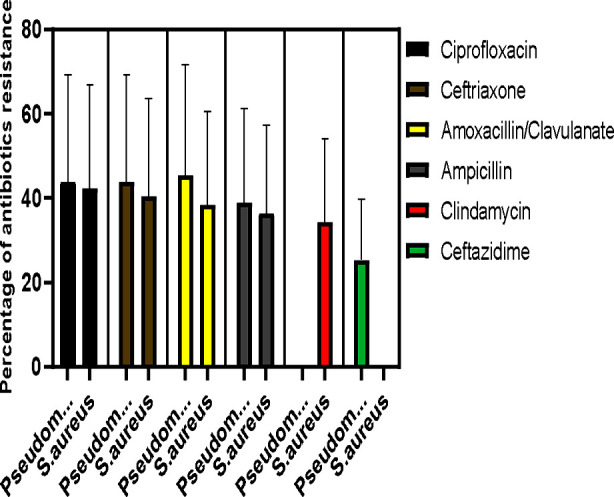
Antibiotic resistance pattern in the two commonest isolated bacteria of CSOM patients.

**Fig.3:**
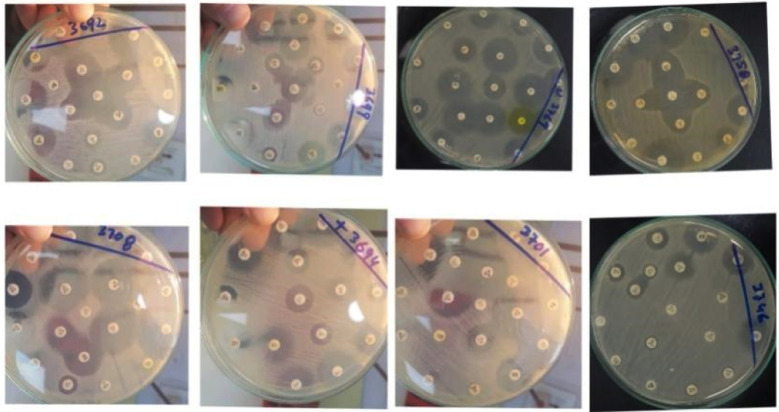
Antibiotic sensitivity pattern of isolated bacteria from CSOM patients on MHA (Mueller Hinton Agar): 3692: E. coli, 3699: Citrobacter, 3769: Serratia marcescens, 3758: Proteus mirabilis, 3701, 3708: Pseudomonas aeruginosa, 3694: Staph aureus, 3746: Enterobacter.

We noted the results as either resistant or susceptible, according to the CLSI guidelines. A well-structured questionnaire was used for collecting patient’s demographic, and clinical data. Patients were followed up after one-month intervals regarding their improvement status and compliance with their antibiotics. Quantitative variables were presented as the number (*n*) and percentage (%). The collected data were assessed by using SPSS software (IBM Corp. Released 2011. IBM SPSS Statistics for Windows, Version 20.0. Armonk, NY: IBM Corp). GraphPad Prism was used for figure making.

## RESULTS

Data was collected from 113 patients. The average age of the patients was 22± 2 years, and 58% were female, with a male-to-female ratio of 1:1.38. Poor educational status was found in 59% and rural residency was found in 68% of individuals (Table-I). Purulent ear discharge was the main symptom (100%), followed by earache (78%), hearing loss (67%), and tinnitus (45%). Out of 113 samples, 97(85.5%) showed growth on culture whereas 16(14%) showed no growth. Out of 97 samples that showed growth, 95 (98%) showed single microbial growth, while 2 (2%) showed growth of multiple microorganisms (one pseudomonas +staph aureus, one pseudomonas+ Coagulase-negative staphylococcus (CoNS)). A total of 99 bacterial isolates were obtained, among the bacteria isolated 66(67%) were gram-negative whereas 33(33%) were gram-positive with Pseudomonas aeruginosa being the commonest bacteria isolated 38(38%) followed by *S. aureus* 25(25%), *Proteus species* 14(14%), *S. saprophyticus* 8(8%), *Providencia* 4(4%), *Citrobacter* 4(4%), *Serratia marcescen*s 2(2%), *Enterobacter species* 2(2%), *E. coli* 2(2%) (Table-II).

**Table-I:** Socio-demographic profile of CSOM patients.

Characteristic	Category	Frequency	Percentage
Age groups	<20 years	17	15
	20-30 years	67	60
	30-40 years	15	14
	>40 years	12	11
Residence	Rural	76	68
	Urban	35	32
Education	Illiterate	66	59
	Primary	18	16
	Intermediate	13	12
	Graduate	8	7
	Postgraduate	5	5

**Table-II:** Percentage distribution of antibiotic sensitivity pattern of bacterial isolates from clinical samples.

Bacteria	AK	GEN	CIP	AMC	AT	PIT	CPM	CFS	CAZ	CTR	IPM	V	LZ	MPM
***Gram Positive***														
Staph aureus		24	16	24		36	16	64	0.08	20	28	76	80	28
CoNS		62	25	25		62	50	87	50	25	75		87	87
***Gram Negative***														
Pseudomonas aeruginosa	55	13	13	10	50	79	55	79	53	13	74			79
Proteus species	64	14	14	28	86	93	50	78	50		93			93
Providencia Species	25	25	25		25	50	50	50	25		50			75
Citrobacter species	25	50	50		25	50	50	50	25		75			100
Serratia marcescens	50					50		50			100			50
Enterobacter species	50										100			100
Escherichia coli	100	100				50		100						50

AK: Amikacin, GEN: Gentamycin, CIP: Ciprofloxacin, AMC: Amoxicillin/Clavulanate, AT: Aztreonam, PIT: Piperacillin tazobactam, CPM: Cefepime, CFS: Cepoferzone/sulbactam, CAZ: Ceftazidime, CTR: Ceftriaxone, IPM: Imipenem, V: Vancomycin, LZ: Linezolid, MPM: Meropenem, TG: Tigecycline. CONS: Coagulase-negative staph.

In terms of antimicrobial susceptibility, among gram-positive bacteria, *S. aureus* was mainly susceptible to Linezolid 80% and Vancomycin 76%. Whereas CoNS were susceptible mainly to Cefoperazone/sulbactam 87%, linezolid 87%, and Imipenem 75%. The resistance spectrum of gram-positive bacteria revealed the following data: Ciprofloxacin (84% to 75%), Ampicillin (70%), Ceftriaxone (75-80%), Amoxicillin/Clavulanate (75%), and Clindamycin (65%).

Meanwhile, the sensitivity spectrum of gram-negative bacteria varied, with susceptibility ranging from 50% to 100% for Imipenem, 50% to 93% for Piperacillin/Tazobactam, 50% to 78% for Cefoperazone plus sulbactam, and 50% to 100% for Meropenem. The resistance profile of gram-negative bacteria, as observed in this study, presented the following data: Ciprofloxacin (50-87%), Ceftriaxone (87%), Ceftazidime (50-75%), Amoxicillin/Clavulanate (72-90%), and Ampicillin (75%).

Regarding follow-up, 93(82%) patients improved after one month of follow-up, among them 78(84%) were compliant with the antibiotics prescribed according to C/S reports, whereas 20(18%) patients were still symptomatic after one month of follow-up, among them 13(65%) were not compliant with medication, showing a significant association of noncompliance of medication with the persistence of symptoms p<0.01 (Table-III).

**Table-III:** Follow-up analysis of patients.

	Improved	Symptomatic	Total	P value
Compliant	78	7	85	<0.001
Non-compliant	15	13	28	

Total	93	20	113	

## DISCUSSION

The study revealed that Pseudomonas aeruginosa was the most commonly isolated bacterium (38%), followed by *S. aureus* (25%). Higher resistance rates were observed against Ciprofloxacin and Ceftriaxone. Gram-positive bacteria showed good susceptibility to Linezolid and Vancomycin, whereas gram-negative to carbapenem, Piperacillin/Tazobactam, and Cefoperazone plus sulbactam. Patient compliance with antibiotic therapy significantly impacted outcomes, with noncompliance strongly associated with persistent symptoms after one month of follow-up

The phenomenon of antibiotic resistance looms ominously, casting a shadow over global health.^6^ In our study, the majority of the patients were from rural areas, with lower education status, and slightly female over presentation, these findings are in line with the literature.^7^ The bulk of the patients presented with purulent discharge from the ear, this matches with the findings of Wan Draman et al.^8^ Single bacteria were isolated from 98% of patients whose culture showed growth, in our study, Akter S et al.^9^ got almost similar results in their study. Predominantly gram-negative bacteria were obtained as compared to gram-positive bacteria (67% vs 33%), and the literature shows similar outcomes.^10^-^11^

The commonest bacteria isolated in our study was *Pseudomonas aeruginosa* which corroborates well with studies.^12^-^13^ The rationale for its chronicity may be its use of pilli for attachment to mucosal surfaces and enzymes for dogging out body defenses. We noted *S.aureus* as the second most common isolate, multiple studies have reported *S.aureus* as one of the leading bacterial causes in the CSOM isolates.^14^-^15^ S. aureus, was followed by *Proteus species*, Coagulase-negative staphylococcus (CoNS), Providencia, *Citrobacter, Serratia*, Enterobacter, and E. coli, which is in agreement with others.^16^ S. aureus was mainly susceptible to Linezolid 80% and Vancomycin 76% whereas CoNS were susceptible mainly to Cefoperazone/sulbactam 87%, linezolid 87%, and Imipenem 75%, other studies have also reported similar effectiveness.^15^-^17^

Against gram-positive causative agents, both vancomycin and linezolid show good activity, though these antibiotics are not considered in routine practice. For gram-negative bacteria, Imipenem, and meropenem, followed by Piperacillin/Tazobactam, and Cefoperazone plus sulbactam, were the effective antibiotics showing their sensitivity from 50 to 100%. The resistance spectrum of gram-negative bacteria showed the following data: Ciprofloxacin (50-87%), Ceftriaxone (87%), Ceftazidime (50-75%), Amoxicillin/Clavulanate (72-90%), and Ampicillin (75%) and our findings align with those of others concerning the susceptibility of gram-negative bacteria.^17^-^19^ Cephalosporins also exhibit decreased sensitivity especially 3^rd^ generation cephalosporins like Ceftazidime and Ceftriaxone, however, the 4^th^ generation cephalosporin Cefepime showed good sensitivity of 50-57%.

Similarly, Ciprofloxacin, a commonly used fluoroquinolone showed less activity with resistance in the range of 50% to 86% against both gram-positive and negative bacteria, this aligns with certain studies but conflicts with others.^20^. Once a drug of choice for chronic suppurative otitis media, to a resistance level of 72-90%, amoxicillin/Clavulanate has suffered resistance the most. One of the systemic reviews and meta-analyses showed a significant degree of resistance against Ampicillin, Amoxicillin/Clavulanate, Cotrimoxazole, Amoxicillin, and Cefuroxime and Our findings are in line with this study.^21^ The escalating antibiotic resistance in chronic ear infections poses a grave concern, reflecting the prevalence of multidrug-resistant (MDR) bacteria. This resistance, spanning various antibiotic classes, underscores the urgency to address the issue. Follow-up studies show that patients who engage in rehabilitation programs demonstrate improved health outcomes, suggesting that ongoing support is beneficial. In contrast, some patients may experience barriers to adherence, such as socioeconomic factors or lack of support, which can hinder effective follow-up and management of CSOM 22.

This study showed new patterns of increasing antibiotic resistance in CSOM, stressing the importance of judicious antibiotic use based on culture results, and patient compliance for improved outcomes.

### Limitations:

The data was collected from a single healthcare setup, limiting the data’s generalizability. The genotypic details of antibiotic resistance of bacteria were not done.

## CONCLUSION

The study concludes that Pseudomonas aeruginosa is the most frequently isolated bacterium. It’s an eye-opener for us to see the development of resistance against antibiotics like ciprofloxacin, third-generation cephalosporin, ampicillin, amoxicillin plus clavulanate, and clindamycin. Linezolid and Vancomycin showed greater efficacy against Gram-positive bacteria, and carbapenem Piperacillin/Tazobactam and Cefoperazone plus sulbactam were effective against Gram-negative bacteria. Moreover, patient compliance with antibiotic therapy was crucial, as noncompliance was significantly associated with the persistence of symptoms, highlighting the importance of adherence to treatment for better clinical outcomes.

## Recommendations:

In countries like Pakistan, factors such as unethical antibiotic prescriptions for minor ailments, unrestricted over-the-counter availability, and limited access to facilities where bacterial culture can be done, exacerbate the problem of antimicrobial resistance (AMR). To combat this challenge effectively, raising awareness about the severity of AMR through targeted educational campaigns is crucial. Moreover, the implementation of antibiotic stewardship programs (ASPs) in tertiary care hospitals is essential to optimize antimicrobial prescribing practices both in inpatient and outpatient settings. These initiatives are imperative to safeguard the effectiveness of antibiotics in treating CSOM and prevent further escalation of antibiotic resistance.
